# Association between thyroid function and disease severity in restless legs syndrome

**DOI:** 10.3389/fneur.2022.974229

**Published:** 2022-08-12

**Authors:** Chaofan Geng, Zhenzhen Yang, Xiumei Kong, Pengfei Xu, Hongju Zhang

**Affiliations:** ^1^Henan University People's Hospital, Henan Provincial People's Hospital, Zhengzhou, China; ^2^Fuwai Central China Cardiovascular Hospital, Henan Provincial People's Hospital, Zhengzhou, China; ^3^Henan University Joint National Laboratory for Antibody Drug Engineering, Kaifeng, China; ^4^Henan Provincial People's Hospital, Zhengzhou University People's Hospital, Zhengzhou, China

**Keywords:** restless legs syndrome, thyroid dysfunction, sleep disorder, TSH, FT3, FT4

## Abstract

**Background:**

Restless Legs Syndrome (RLS) is a common neurological disorder. Growing evidence shows that dopaminergic dysfunction and iron deficiency are associated with the pathogenesis of RLS. Additionally, the dopaminergic system is linked with the hypothalamic-pituitary-thyroid (HPT) axis. Thus, the current study aimed to compare thyroid function between RLS patients and healthy subjects and investigate the associations with clinical characteristics of RLS.

**Methods:**

Serum levels of thyroid hormones were investigated in 102 first-episode drug-naïve RLS patients and 80 matched healthy controls (HCs). Baseline data and clinical characteristics were performed by professional personnel. In addition, multivariate regression was used to analyze the relationship between thyroid function and RLS.

**Results:**

Compared with control group, RLS patients had significantly higher serum thyroid-stimulating hormone (TSH) levels (*p* < 0.001), and higher prevalence of subclinical hypothyroidism [Odds ratio (OR) 8.00; 95% confidence interval (CI) = 3.50–18.30; *p* < 0.001]. The Subclinical hypothyroidism rate (47.1 vs. 10%, *p* < 0.001) in RLS patients was higher than the HCs group. Regression analysis revealed that serum TSH (OR = 1.77; 95% CI = 1.41–2.23; *p* < 0.001) was independently associated with RLS. There was a statistically significant positive correlation between TSH and the Pittsburgh sleep quality index (PSQI) scores (*r* = 0.728, *p* < 0.001), and the International Restless Legs Scales (IRLS) points (*r* = 0.627, *p* < 0.001). Spearman correlation analysis showed that FT_3_ was positive correlated with HAMA_14_ score (*r* = 0.239, *p* = 0.015). In addition, compared with the good-sleeper group, poor-sleeper patients had significantly higher serum TSH levels (*p* < 0.001).

**Conclusion:**

Serum levels of TSH and the prevalence of subclinical hypothyroidism were higher in RLS patients, indicating the imbalance between thyroid hormones (TH) and the dopaminergic system may contribute to the development of primary RLS. Additionally, the TH axis may influence the quality of sleep in RLS patients.

## Introduction

Restless Legs Syndrome (RLS), also known as Willis-Ekbom disease (1), is a common neurological sensory-motor disorder, mainly characterized by strong discomfort of the lower limbs at night or at rest and the irresistible desire to move the legs (2), which is estimated to affect 0.1 ~ 15% of the general population (3). RLS patients also suffer from sleep disturbance and depression (4), which can strongly impact the quality of life (5). Currently, RLS can be divided into idiopathic and secondary forms (6). Alternately, secondary RLS may be related to other medical causes, such as multiple sclerosis, Parkinson's disease (PD), anemia, iron depletion, chronic renal failure, and pregnancy (7). Genetic studies had identified MEIS1 as an RLS-predisposing gene (8, 9), which increases the risk of RLS (10). Although the exact pathophysiology of RLS is not fully understood, there is robust evidence that the neurotransmitter dopamine (DA) and iron deficiency are associated with the pathogenesis of RLS (3).

Previous studies on RLS have revealed a decreased fluorine-18-L-dihydroxyphenylalanine (18F-dopa) uptake in the substantia nigra along with the reduction in presynaptic dopaminergic, providing insight into the role of dopaminergic dysfunction in RLS (11–13). In an autopsy study, compared with controls, the dopamine 2 (D2) receptors in the putamina of idiopathic RLS were decreased, which is also associated with the severity of the disease (14). Numerous studies have also shown that DA agonists can alleviate RLS symptoms (15). These findings indicated dopaminergic dysfunction in RLS patients. Interestingly, there are signs that the dopaminergic system is may be associated with the hypothalamic-pituitary-thyroid (HPT) axis (16). Specifically, DA can downregulate thyroid hormones (TH) and thyroid stimulating hormone (TSH) while upregulating-thyrotropin releasing hormone (TRH) (17). Therefore, based on these findings, we hypothesized that the imbalance between thyroid hormones and the dopaminergic system may contribute to the development of RLS.

Currently, on the one hand, it has been reported that the daily pattern of TSH levels is similar to the fluctuation of symptoms in RLS patients (16). On the other hand, the relationship between thyroid function and RLS has been less studied, and inconsistency in conclusions. There are no studies that associate thyroid function and symptom severity of RLS. Therefore, to explore this connection further, the current study aimed to compare thyroid function between RLS patients and healthy subjects, and to investigate the associations with clinical characteristics and symptom severity of RLS, which may contribute to the development of therapeutic strategies for managing and preventing RLS.

## Materials and methods

### Research subjects

Our cross-sectional cohort study was conducted from January 2018 to October 2021, including 102 patients with RLS who were recruited from the neurology department of the Henan Provincial People's Hospital. Additionally, during the same period, 80 sex- and age-matched healthy subjects screened from the healthy examination center of our hospital were selected as healthy controls (HCs).

The study was approved by the Research Ethics Committee of Henan Provincial People's Hospital according to the principles of the Helsinki Declaration. Additionally, all the participants signed the written informed consent forms.

### Inclusion criteria

The patients met the diagnostic criteria of RLS according to the International Restless Legs Syndrome Study Group (IRLSSG) (18). To make the diagnosis, the four essential features were confirmed by the interview. Additionally, to avoid interference from DA agonist treatment, we included subjects who were first-episode drug-naïve RLS patients.

### Exclusion criteria

Studies that met in the following criteria were excluded: (1) RLS secondary to chronic kidney disease, diabetes, rheumatoid arthritis, renal failure, iron deficiency, peripheral neuropathy, PD, drug-induced factors; (2) combined with other sleep disorders such as narcolepsy, rapid eye movement sleep behavior disorder (RBD), narcolepsy, or obstructive sleep apnea syndrome (OSAS); (3) patients with a history of thyroidectomy, neck radiotherapy, ^131^I treatment for hyperthyroidism, or current consumption of antithyroid drugs were also excluded; (4) history of mental disorders (e.g., schizophrenia) or family history of mental disorders; (5) pregnant and lactating women.

### Research methods

#### Collecting general information

Self-reported general information included: gender, age, the age of onset, disease duration (years), and education duration. Their body mass index (BMI), heart rate, and blood pressure were directly measured.

#### Clinical and neuropsychological assessment

For all patients who met the diagnostic criteria, we used the International Restless Legs Scales (IRLS) to assess the severity of RLS (19). For the assessment of the disease-specific quality of life, the Restless Legs Syndrome Quality of Life Questionnaire (QoL-RLS) was used by professional physicians (20). In addition, for neuropsychological assessment, the 14-item Hamilton Anxiety Scale (HAMA_14_), and the 24-item Hamilton Depression Rating Scale (HAMD_24_) (21) were used to assess the anxiety and depression symptoms of patients with RLS.

#### Sleep conditions and grouping

The Pittsburgh sleep quality index (PSQI) has been widely used to measure sleep quality over the past month (22), which consists of 23 items. The global points range from 0 to 21. The higher scores, the worse the quality of sleep. According to the PSQI score, subjects can be divided into poor-sleeper (PSQI > 5) group and good-sleeper (PSQI ≤ 5) group (22).

#### Laboratory assessment

All subjects fasted overnight for 12 h and 5 ml venous blood samples were collected in serum separator tubes and immediately centrifuged at 3 000 r/min and stored at −80° C. According to standard operating procedures, serum TSH, free triiodothyronine (FT_3_), and free thyroxine (FT_4_) levels were measured using electrochemiluminescence immunoassays. In addition, anti-thyroid peroxidase antibodies (anti-TPO) and anti-thyroglobulin antibodies (anti-TG) were also detected. In our hospital, the reference value of TSH, FT_3_, and FT_4_ has been estimated at 3.1–6.8, 12.0–22.0 pmol/L, and 0.27–4.2 uIU/mL, respectively.

Additionally, high TSH and normal FT4 can be diagnosed with subclinical hypothyroidism; High TSH and low FT4 were diagnosed with primary hypothyroidism (23).

### Statistical analysis

For data processing and statistical analysis, the SPSS Statics 24.0 computer software was conducted. Continuous variables were presented as mean ± standard deviation (SD) or median and interquartile range (M, P_25_, P_75_) based on the normal or non-normal distribution of the data determined by the Kolmogorov-Smirnov test. For normally distributed continuous data, we used the student's *t*-test, and for non-normally distributed continuous variables, we used the Mann-Whitney *U*-test. The Chi-square test or Fisher's exact test was used to compare the groups. Categorical data are expressed in amount (%). In order to identify predictive indicators of RLS, a multivariate logistic regression analysis was performed, adjusting for disease duration sex, age, BMI, SBP, and DBP. To compare the prevalence of thyroid dysfunction between RLS patients and controls, the odds ratio (OR) and 95% confidence interval (CI) were calculated. The two–by–two frequency table was performed to calculate OR. The associations between thyroid function and clinical characteristics in RLS were analyzed by Spearman or Pearson. *P* < 0.05 were considered statistically significant.

## Results

### Baseline characteristics

A total of 102 RLS patients (70 female; mean age, 51.58 ± 6.79 years), and 80 HCs (22 female; mean age, 50.86 ± 6.15 years) were recruited for this study. Differences in age, gender, BMI, smoking, and alcohol consumption were not significant between the RLS group and the HCs group (all *p* > 0.05). The Subclinical hypothyroidism rate (47.1 vs. 10%, *p* < 0.001) in RLS patients was higher than those in the HCs group. However, no patients with hypothyroidism were found in the two groups. As compared with the HCs group, serum TSH was significantly higher in the RLS group (*p* < 0.001). Additionally, there was no statistically significant different between both groups in either FT_4_ or FT_3_ (all *p* > 0.05). The demographic and clinical characteristics of all subjects are shown in Table 1.

**Table 1:** Demographic features of control and RLS groups.

**Variables**	**RLS (*n* = 102)**	**Control (*n* = 80)**	***p*-value**
Gender (male/female)	32/70	22/58	0.994
Age (years)	51.58 ± 6.79	50.86 ± 6.15	0.463
BMI (kg/m^2^)	23.83 ± 2.17	24.03 ± 2.02	0.530
SBP (mmHg)	126.35 ± 7.89	128.16 ± 6.70	0.103
DBP (mmHg)	79.79 ± 8.43	79.08 ± 7.76	0.555
Alcohol [*n* (%)]	30 (29.4%)	20 (25.0%)	0.508
Smoking [*n* (%)]	28 (27.5%)	19 (23.8%)	0.571
Subclinical hypothyroidism, *n* (%)	48 (47.1%)	8 (10.0%)	**<0.001**
Hypothyroidism, *n* (%)	None	None	–
TSH (uIU/mL)	3.89 ± 1.83	2.56 ± 1.15	**<0.001**
FT_4_ (pmol/L)	14.27 ± 2.19	14.64 ± 1.72	0.224
FT_3_ (pmol/L)	4.23 ± 0.58	4.35 ± 0.58	0.148
Serum iron (μmol/l)	13.17 ± 0.21	13.21 ± 0.24	0.641
Ferritin (ng/mL)	222.22 ± 0.27	224.71 ± 0.12	0.073
PSQI, points	7 (5, 12)	–	–
HAMA_24_, points	18 (17, 21)	–	**–**
HAMD_14_, points	12 (8, 14)	–	–
IRLS, points	12 (10, 18)	–	**–**
QoL-RLS, points	33 (29, 34)	–	–
Disease duration (years)	3.30 (3.00, 3.85)	–	**–**

BMI, body mass index; SBP, systolic blood pressure; DBP, diastolic blood pressure; PSQI, Pittsburgh Sleep Quality Index (PSQI) scale, HAMD_24_, 24-item Hamilton Depression Rating Scale; -HAMA_14_, 14-item Hamilton Anxiety Scale; IRLS, international restless legs syndrome rating scale; QoL-RLS, the Restless Legs Syndrome Quality of Life Questionnaire. Data are presented as mean ± standard deviation, or median (interquartile range) as appropriate. The differences were considered significant if p < 0.05.

The bold values indicate the value of p < 0.05.

### The prevalence of the thyroid dysfunction

According to the TSH, FT_3_, and FT_4_ levels, 47.1% of the patients (*n* = 48) in the RLS group with high TSH and normal FT4 were diagnosed with subclinical hypothyroidism, whereas, in the HCs group, 10% (*n* = 8) of the subjects with subclinical hypothyroidism. In addition, there was no subject with hypothyroidism (high TSH and low FT4). Patients with RLS were up to 8 times more likely to develop thyroid dysfunction compared to the HCs group (OR = 8.00, 95% CI = 3.50–18.30, *p* < 0.001; Table 2).

**Table 2:** The prevalence of the thyroid dysfunction in RLS.

**Variables**	**RLS (*n* = 102)**	**Control (*n* = 80)**
**Thyroid dysfunction**
Yes	48	8
No	54	72
OR (95% CI)	8.00 (3.50,18.30)	
*P*-value	**<0.001**	

OR, odds ratio; CI, confidence interval.

The bold values indicate the value of p < 0.05.

### Logistic regression analysis

The results of the multiple stepwise logistic regression analysis were shown in Table 3. After adjusting for disease duration sex, age, BMI, SBP, and DBP, serum TSH was independently associated with RLS (OR = 1.77; 95% CI = 1.41–2.23; *p* < 0.001).

**Table 3:** A multiple stepwise logistic regression to identify independent factors of RLS.

**Variables**	**OR (95% CI)**	***p*-value**
TSH	1.77 (1.41, 2.23)	**<0.001**
FT_4_	0.93 (0.78, 1.01)	0.388
FT_3_	0.62 (0.35, 1.08)	0.092

OR, odds ratio; CI, confidence interval; FT3, free triiodothyronine; FT4, free thyroxine; TSH, thyroid-stimulating hormone. Adjusted for age, sex, BMI, SBP, DBP, and disease duration. The bold values indicate the value of p < 0.05.

### Correlation analysis

A significant positive correlation was found between PSQI scores and serum TSH levels in RLS patients by Spearman correlation analysis (*r* = 0.728, *p* < 0.001). In addition, there was also a statistically significant positive correlation between TSH serum level and IRLS points (*r* = 0.627, *p* < 0.001). Spearman correlation analysis showed that FT_3_ was positive correlated with HAMA_14_ score (*r* = 0.239, *p* = 0.015; Table 4).

**Table 4:** Correlation between thyroid hormones and clinical characteristics of RLS.

**Variables**	**TSH**		**FT**_**4**_		**FT**_**3**_	
	***r*-value**	***p*-value**	***r*-value**	***p*-value**	***r*-value**	***p*-value**
PSQI score	0.728	**<0.001**	−0.129	0.195	0.085	0.396
HAMD_24_ score	0.077	0.442	0.042	0.672	0.160	0.107
HAMA_14_ score	−0.074	0.461	0.083	0.405	0.239	**0.015**
IRLS, points	0.627	**<0.001**	−0.015	0.880	0.038	0.708
QoL-RLS, points	0.043	0.665	0.111	0.267	−0.027	0.786

TSH, thyroid stimulating hormone; FT3, free triiodothyronine; FT4, free thyroxin; ESS, Epworth Sleepiness Scale; PSQI, Pittsburgh Sleep Quality Index (PSQI) scale, HAMD_24_, 24-item Hamilton Depression Rating Scale; HAMA_14_, 14-item Hamilton Anxiety Scale; IRLS, international restless legs syndrome rating scale; QoL-RLS, the Restless Legs Syndrome Quality of Life Questionnaire. The bold values indicate the value of p < 0.05.

### Subgroup analysis by sleep disorder

One hundred two patients with RLS, were divided into poor-sleepers (PSQI points > 5) and good-sleepers (PSQI points ≤ 5) groups. As compared with the good-sleepers group, the serum TSH level was higher in the poor-sleepers group (*p* < 0.001). No significant difference was found in FT_4_ or FT_3_ between the two groups (*p* > 0.05; Table 5).

**Table 5:** Serum levels of FSH, FT_4_, and FT_3_ of RLS by PSQI score.

**Variables**	**Poor-Sleepers group (*n* = 49)**	**Good-Sleepers group (*n* = 53)**	***p*-value**
TSH (uIU/mL)	5.18 ± 1.53	2.71 ± 1.17	**<0.001**
FT_4_ (pmol/L)	14.10 ± 2.23	14.44 ± 2.17	0.444
FT_3_ (pmol/L)	4.24 ± 0.52	4.22 ± 0.63	0.862

Poor-sleepers (PSQI points > 5); good-sleepers (PSQI points ≤ 5); PSQI, Pittsburgh Sleep Quality Index; FT3, free triiodothyronine; FT4, free thyroxine; TSH, thyroid-stimulating hormone. The bold values indicate the value of p < 0.05.

## Discussion

To the best of our knowledge, our study is the first to investigate the associations between clinical characteristics and thyroid function among RLS patients. Our study found that serum TSH levels and prevalence of subclinical hypothyroidism were higher in RLS patients, compared with the control group. Regression analysis revealed that serum TSH was independently associated with RLS. Our further spearman correlation analysis found that serum TSH was positively correlated with the PSQI scores and the IRLS points. In addition, compared with the poor-sleeper group, good-sleeper patients had significantly higher serum TSH levels.

### DA system and iron deficiency and HPT axis

Increasing evidence has indicated that dopaminergic dysfunction plays a vital role in RLS pathophysiology (24). There are many studies had showed that DA agonists can be beneficial to RLS patients (15). Evidence indicated that iron deficiency is associated with the pathophysiology of RLS (25).

Studies have shown that iron is involved in processes of DA synthesis, which is a cofactor of tyrosine hydroxylase (26). Similarly, there is evidence that iron deficiency can increase TH levels (27). Notably, it is known that DA depresses the TH axis (28). On the other hand, TRH can be able to stimulate DA release by activating presynaptic dopaminergic neurons (17). Patients with typical RLS have the most severe symptoms at night (29). Interestingly, a previous study found that TSH secretion pattern varies in a circadian rhythm (30), which coincides with the diurnal fluctuations in symptom worsening among RLS patients (16).

### RLS and thyroid function

Currently, the link between hypothyroidism and RLS has been less studied, and the conclusions were inconsistent. There are many studies indicating that patients with pregnancy and Graves' disease had higher levels of TH, which may contribute to the higher prevalence of RLS symptoms (31). A study including 600 pregnant women by Ozer et al. (32) revealed that the prevalence of hypothyroidism was higher in pregnant women with RLS compared to those without RLS (*p* < 0.05), which was consistent with another finding (33). Tan et al. (34) found that there was no significant difference in the prevalence of RLS between thyroid disorders patients and normal individuals. On the contrary, a case–control study of 353 RLS by Ahmed et al. (33) showed that RLS patients had an increased prevalence of hypothyroidism than the healthy controls. Our results also showed an elevated prevalence of subclinical hypothyroidism in RLS patients and serum TSH was independently related to RLS. Considering these findings, thyroid dysfunction can influence RLS symptoms despite the lack of direct evidence that it directly causes RLS. Since thyroid dysfunction is clinically easier to detect as well as treat, it should be taken seriously given that it may be a potentially modifiable risk factor for RLS.

Epidemiological studies have found that, compared with the male population, the prevalence of RLS was higher in the female population (35, 36), which was consistent with our finding that the ratio of female to male RLS patients was 2:1 in our paper. Currently, few studies have elucidated the pathological mechanisms underlying sex-specific differences in RLS patients. According to subgroup analysis by sex, we found no difference in serum TSH, FT_3_, and FT_4_ levels between male and female RLS patients, which we speculate may be associated with the relatively small sample size.

In this study, we found a statistically significant positive correlation between TSH serum levels and IRLS points. Although, we have no direct evidence of a correlation between serum TSH levels and iron levels as well as DA levels. However, we can speculate that the abnormal correlation may exacerbate RLS symptoms.

### Thyroid dysfunction and sleep disorders

There are many studies indicating that hypothyroidism may affect sleep quality (31). A Population-Based Study of 2,224 patients with subclinical hypothyroidism by Song et al. (37) found that patients with subclinical hypothyroidism typically had shorter sleep duration, longer sleep latency, and higher PSQI scores compared to 12,622 controls with normal thyroid function. On the contrary, Akatsu et al. (38) showed that there was no significant association between sleep quality and subclinical hypothyroidism or hyperthyroidism patients. However, the pathophysiological mechanism between hypothyroidism and insomnia is not known.

Epidemiological studies have shown that the incidence of sleep disorders was higher in RLS patients than in the general population (4, 39). In this study, there was also a statistically significant positive correlation between TSH serum levels and PSQI scores. We speculate that sleep quality is closely related to serum TSH levels in RLS patients. Additionally, this study also found that the serum levels of TSH were higher in the poor-sleepers group as compared to the good-sleepers group, indicating a possible association between thyroid dysfunction and sleep disorders.

### Limitations and recommendations

Our study has several limitations. First, our study could not clarify the causal relationship between thyroid dysfunction and RLS, which was a cross-sectional descriptive study. Future large-scale cohort studies should be conducted to confirm the causal relationship. Second, we did not quantify TH with dopamine receptors in the brain, but only used a scale to measure the severity of the disease. Third, we assessed the sleep quality of RLS patients using only the PSQI scale and did not correlate TH with the arousal index (AI) in objective polysomnography to improve the accuracy of our conclusions. Finally, we included all patients with idiopathic first-episode drug-naïve RLS, and the effects of DA agonists on patients' thyroid hormones after their use for treatment should be compared in future studies.

## Conclusion

In conclusion, our results showed that serum levels of TSH and the prevalence of subclinical hypothyroidism was higher in RLS patients, indicating the imbalance between thyroid hormones and the dopaminergic system may contribute to the development of primary RLS. In addition, more research evidence is needed to investigate whether the TH axis may affect sleep quality in patients with RLS.

## Data availability statement

The raw data supporting the conclusions of this article will be made available by the authors, without undue reservation.

## Ethics statement

The studies involving human participants were reviewed and approved by the Research Ethics Committee of Henan Provincial People's Hospital. The patients/participants provided their written informed consent to participate in this study. Written informed consent was not obtained from the individual(s) for the publication of any potentially identifiable images or data included in this article.

## Author contributions

CG and ZY: wrote first draft and statistics. ZY and XK: statistics and data collection. CG and HZ: conceptualization, resources, and supervision. All authors approved the submitted version.

## Funding

This work was supported by the Henan Medical Science and Technology Research Program (No. 202102310082), and Henan Province Medical Science and Technology Tackling Provincial Ministry Key Projects (SBGJ202102033).

## Conflict of interest

The authors declare that the research was conducted in the absence of any commercial or financial relationships that could be construed as a potential conflict of interest.

## Publisher's note

All claims expressed in this article are solely those of the authors and do not necessarily represent those of their affiliated organizations, or those of the publisher, the editors and the reviewers. Any product that may be evaluated in this article, or claim that may be made by its manufacturer, is not guaranteed or endorsed by the publisher.
